# Sleep Loss Reduces the DNA-Binding of BMAL1, CLOCK, and NPAS2 to Specific Clock Genes in the Mouse Cerebral Cortex

**DOI:** 10.1371/journal.pone.0026622

**Published:** 2011-10-24

**Authors:** Valérie Mongrain, Francesco La Spada, Thomas Curie, Paul Franken

**Affiliations:** 1 Center for Integrative Genomics, University of Lausanne, Lausanne, Switzerland; 2 Center for Advanced Research in Sleep Medicine, Hôpital du Sacré-Coeur de Montréal, Department of Psychiatry, Université de Montréal, Montréal, Canada; Yale School of Medicine, United States of America

## Abstract

We have previously demonstrated that clock genes contribute to the homeostatic aspect of sleep regulation. Indeed, mutations in some clock genes modify the markers of sleep homeostasis and an increase in homeostatic sleep drive alters clock gene expression in the forebrain. Here, we investigate a possible mechanism by which sleep deprivation (SD) could alter clock gene expression by quantifying DNA-binding of the core-clock transcription factors CLOCK, NPAS2, and BMAL1 to the *cis*-regulatory sequences of target clock genes in mice. Using chromatin immunoprecipitation (ChIP), we first showed that, as reported for the liver, DNA-binding of CLOCK and BMAL1 to target clock genes changes in function of time-of-day in the cerebral cortex. Tissue extracts were collected at ZT0 (light onset), −6, −12, and −18, and DNA enrichment of E-box or E'-box containing sequences was measured by qPCR. CLOCK and BMAL1 binding to *Cry1*, *Dbp*, *Per1*, and *Per2* depended on time-of-day, with maximum values reached at around ZT6. We then observed that SD, performed between ZT0 and −6, significantly decreased DNA-binding of CLOCK and BMAL1 to *Dbp*, consistent with the observed decrease in *Dbp* mRNA levels after SD. The DNA-binding of NPAS2 and BMAL1 to *Per2* was also decreased by SD, although SD is known to increase *Per2* expression in the cortex. DNA-binding to *Per1* and *Cry1* was not affected by SD. Our results show that the sleep-wake history can affect the clock molecular machinery directly at the level of chromatin binding thereby altering the cortical expression of *Dbp* and *Per2* and likely other targets. Although the precise dynamics of the relationship between DNA-binding and mRNA expression, especially for *Per2*, remains elusive, the results also suggest that part of the reported circadian changes in DNA-binding of core clock components in tissues peripheral to the suprachiasmatic nuclei could, in fact, be sleep-wake driven.

## Introduction

Sleep loss has a profound, negative impact on cognition, learning, mood, and diverse aspects of mental health [1]. Although chronic sleep debt is a growing problem in our society, the molecular and cellular sources of its deleterious effects remain poorly understood. Sleep is known to be regulated by the tight interaction between two main processes [2], [3]: a sleep homeostat that tracks sleep need according to the duration of time spent awake and asleep, and a circadian timing system that determines the propensity for wakefulness according to an about 24 h rhythm.

At the molecular level, the regulation of the circadian timing system has been extensively studied and it is now well-established that circadian rhythms originate from interacting transcriptional-translational feedback loops involving clock genes and their protein products [4]. In contrast, the molecular wiring of the sleep homeostat remains to be defined. The function of the core clock transcription factors CLOCK, NPAS2, and BMAL1 resides in their hetero-dimerization and binding to E-box (CANNTG) or E-box like (E'-box) elements of target genes from which transcription is initiated [5], [6]. Some specific targets such as *Period* (*Per*)*-1, and -2*, and *Cryptochrome (Cry)-1,* and *-2* genes subsequently provide negative feedback by interfering with the CLOCK::BMAL1 or NPAS2::BMAL1 transcriptional complexes thereby inhibiting their own transcription [4]. In addition, other target genes such as the orphan nuclear receptor families *Rev-Erb* and *Ror*, respectively suppress and promote the expression of *Npas2, Bmal1*, and *Cry1*. These feedback loops, in combination with diverse post-translational mechanisms, result in a rhythmic (about 24 h) expression of clock genes, of their protein product, and of a number of output genes (or clock controlled genes) governing a multitude of physiological functions.

It was initially reported that rhythmic gene expression did not depend on rhythmic binding of CLOCK and BMAL1 to the E-box element of a target gene, that of *Per1* in particular [7]. More recent reports clearly demonstrate, however, that CLOCK and BMAL1 bind to clock genes, notably to *Per1*, *Per2*, *Cry1*, *Cry2*, and *Dbp (D site albumin promoter binding protein)*, in a time-of-day dependent manner in the mouse liver [6], [8]–[10]. Similar observations of rhythmic chromatin binding were reported also for the core clock transcription factors CLOCK and CYCLE in the fruit fly, where they were shown to contribute to rhythmic gene expression [11]–[13]. These data imply that, at least for some clock genes, changes in binding of the core clock transcription factors to their genomic sequence play a role in the circadian modulation of their expression.

Work from our group and that of others demonstrate that, in addition to their well established role in generating circadian rhythms, clock genes also play a circadian-independent role in sleep homeostasis [14]. First, mutations in some clock gene change the electroencephalographic (EEG) and molecular markers of sleep pressure in mice [15]–[19]. For instance, *Npas2* ^−/−^ mice show an attenuated EEG delta power response to sleep deprivation (SD), revealed between 1 and 2 Hz, compared to wild-type littermates [19], whereas this marker of sleep intensity is greatly increased during normal sleep in *Cry1,2* ^−/−^ double-knockout mice [16]. Second, an increase in sleep pressure achieved by SD changes the expression of several clock genes in the forebrain of various inbred mouse strains, notably that of *Per1*, *Per2* and *Dbp* [16], [20], [21]. Moreover, although we found that in the mouse some of these sleep/wake-dependent changes, especially those of *Per1* and *Per3*, were driven by the corticosterone surge associated with the SD, the increase in *Per2* expression and the decrease in *Dbp* expression were largely independent of corticosterone [22]. This last finding suggests that the SD-induced changes in the expression of these two clock genes are likely caused by modifications in the activity of the core clock transcription factors (i.e., CLOCK, NPAS2, and BMAL1) upon their respective promoter. The fact that the increase in *Per2* in the brain is reduced in *Npas2^−/−^* mice [19] and increased in *Cry1,2* double KO mice [21] further supports such notion.

We here investigate the potential role of changes in DNA binding activity of core clock transcription factors in the modulation of clock gene expression observed with elevated sleep pressure. Specifically, we hypothesize that SD modifies the binding of clock transcription factors to the E-box and/or E'-box sequences of specific clock genes, especially that of *Per2* and *Dbp*, in the mouse cerebral cortex. Using chromatin immunoprecipitation (ChIP) of cortical tissue, we first observed that the binding of clock transcription factors to several clock genes depended on time-of-day in the mouse cortex in a way comparable to the time course previously described for liver [6], [9], [10]. Importantly, we found that SD alters the binding of clock transcription factors to their target genes. This effect was both transcription factor and target gene specific. Our findings thus reveal that sleep pressure, in addition to internal time-of-day, can modify the DNA-binding properties of core clock proteins in the brain, providing a mechanism by which clock components can sense homeostatic sleep need.

## Results and Discussion

### Rhythmic binding of BMAL1 and CLOCK to selected clock genes

**Figure 1:**
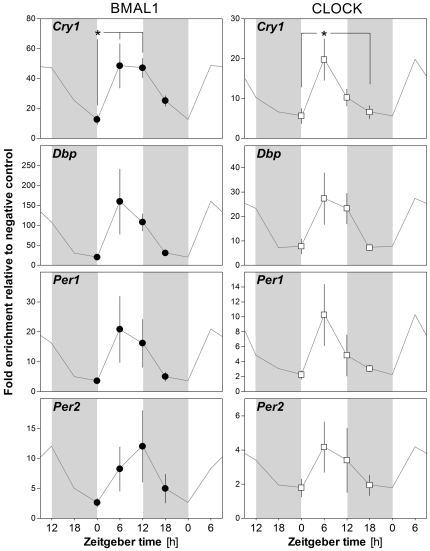
The effect of time-of-day (ZT) on BMAL1 and CLOCK binding onto the promoter of 4 clock genes in the mouse liver. Mice were sacrificed every 6 hours for 24 hours (ZT0 = Lights ON), and livers were rapidly sampled and processed. Chromatin immunoprecipitation (ChIP) was performed using antibodies against BMAL1 and CLOCK, and enrichment of E-box or E'-box containing sequences of the putative promoter region of *Cry1*, *Dbp*, *Per1*, and *Per2* was measured using quantitative PCR. Data were normalized to a negative control condition (no-antibody). Time-of-day significantly affected the binding of BMAL1 (F_3,19_ = 4.3, p<0.05) and that of CLOCK (F_3,21_ = 4.1, p<0.05) to *Cry1*. Similar trends were also observed regarding binding to *Dbp* (BMAL1: F_3,19_ = 2.4, p = 0.1, n.s.; CLOCK: F_3,19_ = 2.7, p = 0.08, n.s.) and CLOCK binding to *Per1* (F_3,21_ = 2.0, p = 0.1, n.s.). Data are presented as mean ± SEM (n = 5, 5, 5, 5 for all genes for BMAL1; *Cry1* n = 5, 6, 5, 6; *Dbp* and *Per2* n = 5, 5, 5, 5; *Per1* n = 6, 6, 5, 5 for CLOCK for ZT0, −6, −12, and −18, respectively). Grey lines connect double-plotted data, and light grey areas represent 12 h dark periods. (*: p≤0.05 between indicated points; post-hoc Tukey comparisons).

We have previously shown that SD does not uniformly affect the expression of all clock genes in the brain [16], [19]–[23]. For the present chromatin binding study, we selected *Cry1* which shows either no change or a small amplitude decrease or increase following SD, *Dbp* for which mRNA consistently decreases after SD, and *Per1* and *Per2* which mRNA are increased by SD. To validate our technique, we first confirmed rhythmic binding of CLOCK and BMAL1 to the E-box or E'-box elements of the promoter of the four selected clock genes in the mouse liver. Livers collected at 4 different times of day (ZT0, −6, −12, and −18) were processed using ChIP. Consistent with the literature [6], [8]–[10], we observed that the binding of BMAL1 and CLOCK to *Cry1* and *Dbp* was higher at ZT6 (i.e., 6 h after lights on) than at ZT18 (i.e. 6 h after lights off) or ZT0 (Figure 1). A similar phase was observed for BMAL1 and CLOCK binding to *Per1* and *Per2* promoters (Figure 1).

**Figure 2:**
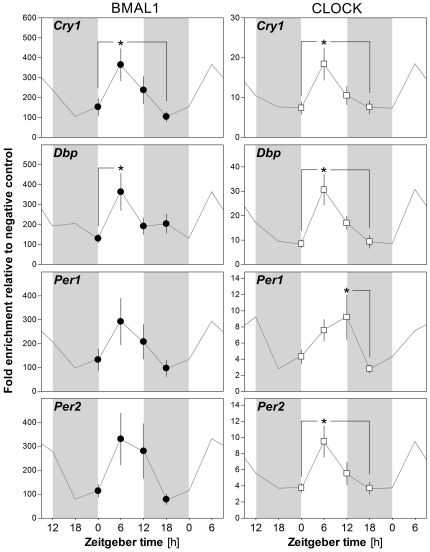
The effect of time-of-day (ZT) on BMAL1 and CLOCK binding onto the promoter of 4 clock genes in the mouse cerebral cortex. Mice were sacrificed every 6 hours for 24 hours (ZT0 = Lights ON), and cerebral cortices were rapidly sampled and processed. Chromatin immunoprecipitation (ChIP) was performed using antibodies against BMAL1 and CLOCK, and enrichment of E-box or E'-box containing sequences of the putative promoter region of *Cry1*, *Dbp*, *Per1*, and *Per2* was measured using quantitative PCR. Data were normalized to a negative control condition (no-antibody). Time-of-day significantly affected the binding of BMAL1 to *Cry1* and *Dbp* genes, and the binding of CLOCK to all 4 target genes. Data are presented as mean ± SEM (*Cry1* n = 14, 16, 14, 13; *Dbp* n = 14, 15, 13, 15; *Per1* n = 16, 16, 14, 15; *Per2* n = 16, 18, 17, 17 for BMAL1; *Cry1* n = 13, 13, 12, 11; *Dbp* n = 12, 14, 11, 13; *Per1* n = 15, 14, 12, 14; *Per2* n = 14, 15, 15, 16 for CLOCK for ZT0, −6, −12, and −18, respectively). Grey lines connect double-plotted data, and light grey areas represent 12 h dark periods. (*: p≤0.05 between indicated points; post-hoc Tukey comparisons).

Daily changes in chromatin binding of core clock transcription factors to the target genes of interest have not been assessed in the mammalian brain. Therefore, and to put the eventual changes incurred by the SD into context (see below), we determined the time-of-day variations in DNA-binding of BMAL1 and CLOCK to the four selected clock genes in the mouse cerebral cortex. Cortical tissue from the same 4 different times of day (ZT0, −6, −12, and −18) were analyzed using ChIP. The binding of both core clock transcription factors to their four target genes exhibited highly similar time-of-day variations (Figure 2). Specifically, CLOCK bound to the four target genes in a time-of-day-dependent manner with maximum binding reached at ZT6 and lowest binding at ZT18 or -0 for *Cry1* (one-way ANOVAs: F_3,48_ = 3.9, p = 0.01), *Dbp* (F_3,49_ = 6.8, p<0.001), and *Per2* (F_3,59_ = 4.6, p<0.01), while the binding to *Per1* peaked at ZT12 and reached a minimum at ZT18 (F_3,54_ = 3.8, p<0.02). BMAL1 binding showed a similar pattern with maximal levels reached at ZT6 and a minimum at ZT18- or -0 for *Cry1* (F_3,56_ = 4.3, p<0.01) and *Dbp* (F_3,56_ = 2.9, p<0.05), whereas, although similar, the time-of-day changes in *Per1* and *Per2* binding did not reach statistical significance (F_3,60_ = 1.7, p = 0.2, n.s. for *Per1*; F_3,67_ = 2.2, p = 0.09, n.s. for *Per2*).

We here demonstrate, for the first time, that in the cerebral cortex, the binding of BMAL1 and CLOCK to the targeted clock genes varies in function of time of day. These observations are similar to the only other study having assessed BMAL1 binding in the mouse brain [24], which showed a higher binding of BMAL1 to the clock gene *Rev-Erbα* and to *Maoa* at ZT6 than at ZT18. Our data, in particular for *Cry1* and *Dbp*, are also consistent with the observations made in the liver where ChIP for BMAL1 and CLOCK also resulted in a higher level of amplified *Cry1* and *Dbp* promoters at ZT6 compared to ZT18 [6], [8]–[10]. The phase positions of maximum and minimum RNA levels are similar between the brain and liver for the different transcripts targeted (Figure 4 for a review). Therefore, for each target, similar DNA-binding rhythms between the two tissues were expected. We observed a similar phase regarding the binding of clock transcription factors to *Per1* and *Per2* promoters in the cortex. This rhythm seemed, however, less robust as it did not reach statistical significance for BMAL1. In the liver, one study showed that BMAL1 and CLOCK binding to *Per1* promoter did not vary with time-of-day [7], while another study indicated an inverse phase for *Per2* binding by CLOCK (i.e., higher at ZT15 than at ZT6) although binding was not quantified [25]. A recent study indicates, however, that BMAL1 binding to both *Per1* and *Per2* in the mouse liver appears to peak at ZT6 [10], similar to our findings in the cortex. In the fruit fly, a highly rhythmic binding of CLOCK to *Per*, with a peak centered around ZT12, was reported and was shown to be closely linked to the rhythm of *Per* mRNA levels [11]–[13]. Given the variable results obtained in mice for DNA-binding to *Per1* and *Per2*, it is possible that transcriptional rhythms of mammalian *Per* genes depend less on a highly rhythmic DNA-binding of CLOCK and BMAL1 compared to those of *Cry1* and *Dbp* or that of *Per* in *Drosophila*.

### Sleep deprivation decreases BMAL1, CLOCK, and NPAS2 binding to specific clock genes

**Figure 3:**
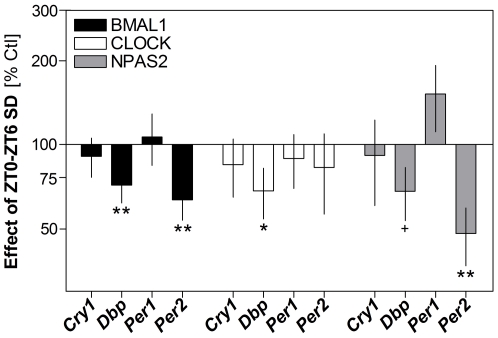
The effect of sleep deprivation (SD) on BMAL1, CLOCK, and NPAS2 binding onto the promoter of 4 clock genes in the mouse cerebral cortex. SD was performed between ZT0 and −6 after which cerebral cortices were rapidly sampled and processed together with non-sleep deprived controls. Chromatin immunoprecipitation (ChIP) was performed using antibodies against BMAL1, CLOCK, and NPAS2, and enrichment of E-box or E'-box containing sequences of the putative promoter regions of *Cry1*, *Dbp*, *Per1*, and *Per2* was measured using quantitative PCR. Data were normalized to a negative control condition (no-antibody) and expressed relative to the control non-sleep deprived sample performed in parallel. SD significantly decreased the binding of BMAL1 to *Dbp* and *Per2*, the binding of CLOCK to *Dbp* gene, and the binding of NPAS2 onto *Per2*. Data are presented as the mean SD/control percentage ratio (± SEM of the ratio; n = 18, 17, 16, 14 for BMAL1; n = 10, 10, 9, 8 for CLOCK; n = 9, 8, 8, 6 for NPAS2 for *Cry1*, *Dbp*, *Per1*, and *Per2*, respectively). Please note logarithmic scaling of y-axis (+: p<0.07, *: p = 0.05, **: p<0.01; t-tests).

To assess the effect of sleep loss on the binding activity of core clock transcription factors to the targeted clock genes, we measured the impact of a 6 h SD performed between ZT0 and −6 on the binding of BMAL1 and CLOCK to the promoter of clock genes in the mouse cerebral cortex (Figure 3). We observed that SD specifically decreased the binding of BMAL1 to the promoter of *Dbp* and *Per2* (t-tests: t = −3.0, p<0.01 and t = −3.7, p<0.01, respectively), whereas binding to *Cry1* and *Per1* genes was not affected by SD (p>0.5, n.s.). The binding of CLOCK to *Dbp* was also significantly decreased by SD (t = −2.2, p = 0.05) while CLOCK binding to the other clock genes assessed (i.e., *Cry1*, *Per1*, and *Per2*) was not affected (p≥0.5, n.s.).

**Figure 4:**
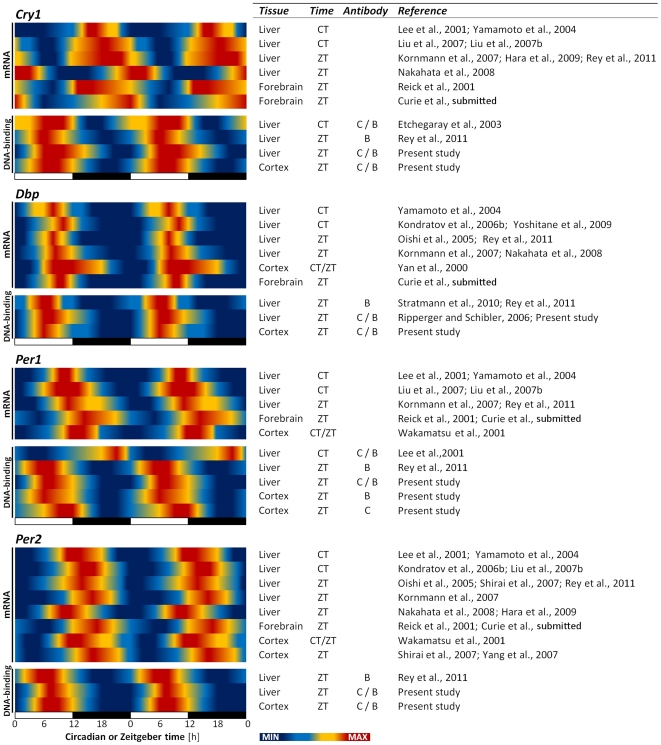
Overview of the phase relationship between RNA expression and chromatin binding. Time-of-day or circadian variations in mRNA expression for the selected clock genes (i.e., *Cry1*, *Dbp*, *Per1*, and *Per2*) for liver and brain samples from selected literature together with variations in DNA-binding of CLOCK (C) and BMAL1 (B) onto these clock genes shown from all previous literature in mice (i.e., liver) and the present study [6]–[10], [45], [62]–[75]. Relative heat scales go from blue (minimum) to red (maximum). ZT: Zeitgeber time with ZT12 = dark onset; CT: Circadian time with CT12 =  activity onset. For *Cry1*, RNA expression and DNA-binding seem to be in antiphase for both liver and brain, whereas the two rhythms are always in-phase for *Dbp*. Regarding *Per1* and *Per2*, the peak in DNA-binding precedes by several hours that of RNA expression for both tissues.

Since BMAL1 binding to *Per2* and not that of CLOCK was decreased by SD, we hypothesized that the SD-mediated change in BMAL1 binding on the promoter of *Per2* could be mediated by the interaction of BMAL1 with its alternative partner, NPAS2. NPAS2, a CLOCK homolog, can associate with BMAL1 and perform a similar role in regulating clock genes transcription [26]. In addition, NPAS2 is predominantly expressed in the brain, particularly in the cerebral cortex and thalamus [27], and was shown to be involved in the response to SD including the increase in EEG slow waves, in NREM sleep duration, and in *Per2* mRNA [19]. We therefore also quantified the effect of SD on the binding of NPAS2 to the chromatin of clock genes in the mouse cerebral cortex (Figure 3, right grey columns). We observed that SD significantly decreased the binding of NPAS2 to *Per2* (t = −4.6, p<0.01). A similar tendency was observed regarding the binding of NPAS2 to *Dbp* (t = −2.2, p<0.07, n.s.), while, again, no change in NPAS2 binding to *Cry1* and *Per1* genes was observed (p>0.2, n.s.).

We thus revealed that elevated sleep pressure changes the binding of the three core clock transcription factors (i.e., BMAL1, NPAS2, and CLOCK) to specific clock genes. As expected, significant SD-dependent changes in DNA binding were observed only for *Dbp* and *Per2*, the two clock genes showing a response mostly independent of the glucocorticoids surge associated with SD [22]. Regarding the DNA binding to *Cry1*, the observed absence of changes following SD is consistent with the lack of a clear SD effect upon *Cry1* mRNA expression. Also for *Per1*, we did not observe a change in DNA binding following SD although SD clearly increases *Per1* expression in the mouse brain [16], [20], [22], [23]. We recently showed that this SD-induced increase in *Per1* expression is mediated through a surge in glucocorticoids [22], which combined with the present observations suggest that changes in *Per1* expression following SD are largely due to modifications in its transcription via the activation of a glucocorticoid-response element (GRE) present in its promoter [28], and not through activation involving the core clock components.

Our results specifically show that SD decreases the binding of CLOCK, NPAS2, and BMAL1 to *Dbp* in the mouse brain. Since we previously observed that the SD-dependent decrease in *Dbp* expression was not affected by the glucocorticoid surge [22], and since core clock transcription factors are known to be the main transcriptional regulators of *Dbp* [6], [10], the decreased binding of BMAL1 and CLOCK, and to some extent also that of NPAS2, might be sufficient to explain the decrease in *Dbp* expression after SD. However, the present findings cannot exclude the contribution of other pathways in mediating the SD-dependent decrease in *Dbp* expression. For instance, TNFα was recently shown to decrease *Dbp* expression via a TNFR1 and calcium-dependent pathway [29]. These authors also showed that TNFα could increase *Per1* expression, which could contribute, in combination with a GRE-mediated response [22], to the SD-dependent increase in *Per1*. The TNFα pathway is relevant to sleep regulation as reduced sleep duration was associated with increased TNFα in humans and rodents [30], [31], and inactivation of TNFα or TNFR1/IL1R1 alters sleep amounts and EEG [32], [33]. Hence, core clock transcription factors are most likely not the sole contributors to the SD-dependent changes in clock gene expression, and future studies aiming at assessing the SD-dependent changes in clock gene expression in animals bearing mutations of core clock transcription factors will be required to understand their specific contribution.

The observation of a SD-dependent decrease in NPAS2 binding and a lack of effect of SD upon CLOCK binding on *Per2* was unexpected given the overlapping roles of CLOCK and NPAS2 in the maintenance of circadian rhythmicity in behavior and gene expression [26], [34]. In peripheral tissues, referring here to non-suprachiasmatic nucleus tissues, the presence of CLOCK was shown, however, to be required for the persistence of rhythmic gene expression [35]. The sequence of CLOCK and NPAS2 are similar except for the presence of an extra 43 aa. at the carboxy end of CLOCK. In addition, NPAS2 also lacks an acetyl-CoA binding motif (aa. 656–664) and a poly-glutamine sequence (aa. 753–776) compared to CLOCK. The acetyl-CoA binding motif has been involved in conferring CLOCK with a histone acetyltransferase (HAT) activity [36]. These sequence differences might prevent elevated sleep pressure from changing CLOCK binding to *Per2*, whereas allowing it for *Dbp*. This indicates that, within the same tissue, the effect of elevated sleep pressure on clock gene expression involves complex and different patterns of transcriptional regulation which are highly specific to each clock gene.

We observed that the increase in *Per2* expression following SD [16], [20], [22], [23] was accompanied by a decrease in binding of BMAL1 and NPAS2 to its promoter, which seems paradoxical. It could be argued that the SD-dependent increase in *Per1* and *Per2* mRNA followed by an increase in protein [45] would not initially be driven by BMAL1::CLOCK/NPAS2 but rather, among others, through glucocorticoids::glucocorticoid receptor binding to GRE and/or CREB (cAMP response element binding protein) binding to CRE [37], another pathway that has been linked to sleep regulation [38]. Subsequently, increase in nuclear PER2 could decrease the DNA binding activity of core clock transcription factors and subsequently mRNA levels of their target genes like *Dbp*. This possibility is supported by our observation that *Per1* and *Per2* mRNA levels are increased as early as after 1 h of SD whereas the *Dbp* decrease occurs after 3 to 6 h of SD [20]. To address this possiblity, the detailed time course of core clock transcription factors binding and of glucocorticoid receptors binding to the target DNA regions of *Per1* and *Per2* and their relation to nuclear levels of PER1 and PER2 protein levels will need to be assessed over the course of SD. To further complicate matters, BMAL1 and CLOCK were recently shown to be able to repress glucocorticoids-mediated transcription [39].

### Core clock transcription factor binding and transcriptional activation

To clarify the link between DNA binding and mRNA levels, we examined the relationship between the two in the mouse liver and brain. We first observed that during the time when SD is usually performed (i.e., between ZT0 and −6), the binding of BMAL1 and CLOCK to all clock genes assessed gradually increases during control conditions when animals are mainly asleep (Figures 1 and 2). Increased DNA-binding during this time is not always linked to a mRNA increase of the corresponding gene. For instance, while the expression of *Dbp* is increasing during that interval, the mRNA levels of *Cry1*, *Per1*, and *Per2* are decreasing [20], [23]. The current literature indeed supports a range of phase relationships between the two rhythms (Figure 4). For *Cry1*, the peak in chromatin binding is in antiphase with the peak in mRNA levels for both liver and cortex (about 12 h apart). This contrasts the data regarding *Dbp* for which the peak in chromatin binding and mRNA expression coincide. Regarding *Per1* and *Per2*, there is a phase delay of about 6 h such that when BMAL1 and CLOCK binding begins to decrease, the mRNA levels reach their peak. These observations are consistent with a recent report that assessed both the time-of-day rhythm of BMAL1 binding to these clock genes and the mRNA expression rhythms in the mouse liver [10]. Accordingly, elevated or decreased BMAL1 and CLOCK DNA-binding are not directly linked to increased or decreased mRNA levels, respectively, and this seems especially true for *Cry1*, *Per1*, and *Per2*, and in both the cerebral cortex and liver.

Previous work described several molecular mechanisms likely to contribute to the delay between chromatin binding and gene expression. For instance, it has been suggested that the phase of some transcripts is due to the interplay between several different transcriptional regulators acting on their promoter [40]. Similarly, Rey et al. [10] proposed that the higher the dependence on BMAL1 transcriptional regulation, the smaller the time lag between chromatin binding and gene expression. In particular, REV-ERBα could prevent the recruitment of RNA polymerase II to the *Cry1* promoter at CT6, thereby interfering with CLOCK::BMAL1 transcriptional activation [8]. Furthermore, the CLOCK::BMAL1 complex itself was shown to repress *Cry1* gene expression when CRY1 bound to the complex [41]. In *Drosophila*, the regulation of *Per* and *Tim* expression was shown to depend on the delicate balance between the binding of CLOCK and that of the repressor PER [13], which was suggested to drive two phases of repression: an ON-DNA phase and an OFF-DNA phase. Also other regulators such as the repressor DEC1, can affect *Per* expression [42]. Such mechanisms might be relevant to our observation of a decreased binding to *Per2* simultaneous to an increased mRNA level after SD, and stress the complex transcriptional regulation of clock genes by clock elements in addition to their regulation by non-clock elements (see above). Also, chromatin state importantly contributes to the relationship between DNA-binding and transcriptional activation as transcription is possible only when chromatin is in a permissive configuration. As mentioned earlier, CLOCK is a HAT required for rhythmic expression of *Dbp* and *Per1* independent of DNA-binding [36]. Furthermore, MLL1, a histone methyltransferase, was recently identified as a protein partner of CLOCK and an essential component to both *Dbp* and *Per2* rhythmic expression [43]. These mechanisms are likely to contribute also to the highly specific transcriptional regulation of individual clock genes. This specificity seems to be similarly reflected at the level of the so-called circadian and the sleep pressure-dependent changes, since the various phase relationships between rhythms of chromatin binding and gene expression (antiphase, in-phase, delayed) are also linked to a specific response to SD in terms of mRNA levels (*Cry1* no distinct change, *Dbp* decrease, *Per genes* increase, respectively) and core clock transcription factor binding.

In the present study, the effect of SD on DNA binding was assessed at the time when the binding of core clock transcription factors peaks (i.e., ZT6; Figure 2). This could have biased our results towards emphasizing decreases in binding as opposed to increases. To address this issue one could assess the effects of SD on DNA-binding at different circadian phases. Moreover, it is possible that the SD protocol could have shifted the phase of core clock transcription factors binding to the DNA of their specific targets, namely that of *Dbp* and *Per2*. This could occur either through a direct effect of wakefulness on the cerebral cortex molecular clock machinery or via resetting of the suprachiasmatic nucleus clock, which was shown to respond to behavioral stimulation (arousal and locomotor activity) [44]. Along this line, it is of interest to note that the main normal waking episode in mice, occurring between ZT12 and −18, is also accompanied by a decrease in *Per2* DNA binding by clock transcription factors and increased levels of *Per2* mRNA (Figure 4). Thus, the distribution of sleep and waking under baseline conditions might contribute to the so-called circadian changes in DNA-binding and gene expression in the mouse brain as we recently modeled in detail [45].

### Perspectives and conclusions

Neuronal plasticity and neuroprotection are among the functions likely to be direct correlates of sleep need [22], [23], [46]. Clock genes have been shown to be involved in plasticity, particularly *Per genes* and *Npas2* [47], [48]. For example, impaired hippocampal LTP was observed in *Per2* mutant mice [49], and decreased long-term memory of courtship and waking experience-dependent increase in sleep in *Per* mutant flies [50], [51]. Therefore, clock genes could modulate cortical synchrony and synaptic equilibrium associated with vigilance states, which is further illustrated by the effect of clock-gene mutations on sleep homeostasis [14]. Combining ChIP with high throughput sequencing of cerebral cortex sampled either after sleep or SD could help to identify further targets of BMAL1 and CLOCK/NPAS2 relevant to synaptic plasticity.

The transcriptional response to SD depends on brain area [52], [53]. Also the change in *Per* expression after SD was shown to express a characteristic neuroanatomical pattern, with elevated sleep pressure producing more pronounced increases in the cerebral cortex [20], [21]. In the present study, the SD-dependent changes in chromatin binding were specifically assessed in the cerebral cortex to more closely investigate their role in the recovery aspect of sleep which is known to be linked to EEG activity in slow frequencies [3], [46]. Nonetheless, the role of clock genes could extend to other, non-cortical brain areas. Also, we measured the binding of BMAL1, CLOCK, and NPAS2 to a single E/E'-box containing sequence. It is believed that the number of different E/E'-box containing sites bound by those transcription factor complexes within a gene will strengthen transcriptional control [10]. Accordingly, characterization of the SD effect upon binding to several sites in the promoter and intronic regions of our genes of interest will be useful to clarify the detailed molecular mechanisms governing SD-dependent changes in clock genes expression.

In conclusion, we reported that in the mouse cerebral cortex, core clock transcription factors bind to the DNA of target clock genes in function of time-of-day. Moreover, we revealed that extended wakefulness changes the binding of core clock transcription factors to the promoter of specific clock genes; specifically, SD decreased CLOCK binding to the promoter of *Dbp*, and decreased BMAL1 and NPAS2 binding to *Per2* and *Dbp*. These sleep-wake dependent changes in DNA-binding are likely contributing to the observed alterations in gene expression following SD, and also to changes observed with time-of-day. Together, our data further support a role for clock genes in the homeostatic regulation of sleep. An avenue we are currently following up on, is how elevated sleep pressure changes the multi-clock protein complex associated to the BMAL1::CLOCK/NPAS2 dimers. Also, future research studying the link between clock genes and sleep homeostasis should focus on the involvement of post-translational modifications, for instance on the acetylation status of the core clock proteins that was shown to tightly regulate their function [54], [55].

## Materials and Methods

### Animals and protocol

C57BL/6J mice originally purchased from Jackson Laboratory (Bar Harbor, ME, USA) were bred on site and maintained under standard animal housing conditions (free access to food and water, 12h-light/12h-dark cycle, 23°C ambient temperature). Males between 11 and 15 weeks at the time of the experiments were used in this study. For the time-of-day experiment, mice were sacrificed at ZT0 (Zeitgeber time 0: lights ON), ZT6, ZT12 (lights OFF), and ZT18 by cervical dislocation and the liver and cerebral cortex were immediately removed and fixed (within 1 min), and processed for chromatin extraction. For the sleep deprivation (SD) experiment, mice were sleep-deprived for 6 h starting at ZT0 by gentle handling [56], and sacrificed at ZT6 together with non-sleep-deprived controls. Cerebral cortices were immediately sampled, fixed and processed for chromatin extraction. Experiments were performed with 2 to 4 animals per time (ZT) or condition (SD vs. control), independently repeated 4 to 6 times, and samples were processed randomly. *Ethics statement:* All experiments were approved by the Cantonal Veterinary Office of Vaud.

### Chromatin immunoprecipitation (ChIP)

Chromatin extraction and immunoprecipitation was performed with the Magna ChIP G commercial kit (Millipore Corporation, Billerica, MA, USA) according to the manufacturer's instructions. Briefly, cortices were chopped in small pieces and incubated for 10 min in 1% formaldehyde followed by a 5 min glycin-quenching. After washes and cellular and nuclear lysis, chromatin breakdown was performed with a Bioruptor UCD-200TO (Diagenode SA, Liège, Belgium) to achieve a range of 300–1000 bp chromatin lengths as determined by ethidium bromide agarose gel electrophoresis (cycles 30 sec ON-30 sec OFF at maximum intensity for 15 min). A pre-clear step was performed (1 h at 4°C with 40 µl of magnetic beads) before overnight incubation of chromatin and beads with antibodies. Anti-BMAL1 antibody was from Abcam (Cambridge, England, product #ab3350), and anti-CLOCK from Abcam (product #ab461) or Santa Cruz Biotechnologies (product #sc-6927). These commercial antibodies have been used by independent groups for circadian rhythm research including for ChIP [57]–[59]. Custom anti-NPAS2 antibodies were produced from rabbit immunization using a peptide directed against amino acids 796 to 810 of the C-terminal domain of NPAS2 (accession #NP_032745) by Lucerna-Chem AG (Lucerne, Switzerland). The specificity of this antibody was verified by ELISA and Western blot on brain protein extracts (91 kDa). All samples were also identically submitted to a no-antibody condition (mock condition) used to take into account nonspecific binding. DNA purification was performed using the silica columns provided with the kit (Magna ChIP G, Millipore).

### Quantitative PCR

Quantitative PCR (qPCR) was performed according to Applied Biosystems protocol using a 7900HT Fast Real-time PCR System with SDS2.3 software (Applied Biosystems, Foster City, CA, USA). For each qPCR reaction, purified immunoprecipitated DNA was slightly diluted (4/5) and mixed with 0.9 µM of each primers, 0.25 µM of probe and MasterMix reagent (Applied Biosystems) or FastStart Universal Probe Master (Roche Diagnostic GmbH, Mannheim, Germany). The qPCR was performed under standard cycling conditions (i.e., 95°C for 10 min, followed by 45 cycles of 95°C for 15 sec and 60°C for 1 min). Each qPCR reaction was done in triplicate. Primers and probes were designed with Primer Express v2.0 (Applied Biosystems) to amplify E-boxes and E'-boxes containing genomic regions of the four target clock genes (*Cry1*, *Dbp*, *Per1* and *Per2*). More precisely, the amplicons targeted functional sequences located in the promoter region: most proximal E-box (CACGTG) and E'-box (CACGTT) of *Cry1* (similar to [8], same as [60]), the most proximal E-box (CATGTG) of *Dbp* [6], the first E-box (CACGTG) of *Per1* [5], [37], and two E-boxes (CAGATG, CATTTG) of *Per2* [37]. As an additional negative control, an amplicon targeting a non-CLOCK::BMAL1 target (i.e., exon 5 of *Pmp22*) was also amplified for liver samples and for half of cortical samples (Figures S1 and S2). The oligonucleotide sequences and their source are provided in Table 1. Primers were purchased from Invitrogen or Microsynth (Balgach, Switzerland) and probes from Eurogentec SA (Seraing, Belgium).

**Table 1:** Primers and probes used for qPCR.

Gene	Forward (5′ –>3′)	Reverse (5′ –>3′)	Source
*Cry1*	CCCCGGCTTCTCATTGG	CAGTGTAGTAAACACACTTCAGAAACGT	NCBIM37:10:84593000:84647800:1
probe	FAM-CCCCACGTGACCACCGGCA-BQH		
*Dbp*	ACACCCGCATCCGATAGC	CCACTTCGGGCCAATGAG	NCBIM37:7:52959000:52965561:1
probe	FAM-CGCGCAAAGCCATGTGCTTCC-TAMRA		(see Ripperger and Schibler, 2006)
*Per1*	AGCTTTAGCCACGTGACAGTGA	GGCAAGTGAAGAGGCCAACA	AB030818
probe	FAM-TGCACTTAACAGCTGATTATGTCAGCCGC-BHQ		
*Per2*	AGAAAAGCCCTGCTGTTCCA	CCAAATGCGGTGGTGTAGTTT	AF491941
probe	FAM-CCGCTTCCATAGTTCCTGTAAGGT-BQH		
*Pmp22*	TTCGTCAGTCCCACAGTTTTCTC	ACTCGCTAGTCCCAAGGGTCTA	NM_008885
probe	FAM- CGGTCGGAGCATCAGGACGAGC-BHQ		

### Statistical analysis

Tissue sampled from each animal was treated separately and data from the repeated experiments were combined. Similar to what was performed previously [6], [8]–[10], binding was initially analyzed as a fold relative to input chromatin (Figure S1 for liver and Figure S2 for cerebral cortex). However, we observed that non-specific binding (no-antibody condition) varied and was influenced by time-of-day for liver (Figure S1 legend). Thus, data were expressed in fold-change relative to the no-antibody negative control condition, as performed in other studies [12], [61], in order to control for non-specific binding and also for between-experiment differences. The number of biological replicates (i.e., the number of individual mice) contributing to each data point is indicated in each figure. Numbers vary because of data loss and removal of outliers; i.e., values more than two standard deviations away from the mean (<6% of all values). Fold enrichments were calculated as a ratio of the power of Ct between sample and the respective no-antibody control (i.e., fold enrichment =  [2̂(- Ct sample)]/[2̂(- Ct no-antibody)]). The effect of time-of-day on chromatin binding was assessed by one-way ANOVA, and significant effects were decomposed using Tukey tests. For assessing the SD effect on chromatin-binding, between-experiment differences were taken into account by expressing each SD enrichment level in percent of the enrichment level of a non-sleep deprived control processed in the same batch ([fold enrichment SD/fold enrichment control]*100). The effect of SD was then analyzed using two-tailed single sample t-tests. Statistical analyses were performed with GraphPad Prism 4 (GraphPad Software Inc., San Diego, CA) or Statistica (Statsoft Inc., Tulsa, OK). The threshold for statistical significance was set to<0.05 and results are reported as mean ± SEM.

## Supporting Information

Figure S1 **BMAL1 and CLOCK binding onto the promoter of 4 clock genes in the mouse liver.** Mice were sacrificed every 6 hours for 24 hours, and livers were rapidly processed. Chromatin immunoprecipitation (ChIP) was performed using antibodies against BMAL1 and CLOCK or no antibody (negative control), and enrichment of putative promoter sequences of *Cry1*, *Dbp*, *Per1* and *Per2*, and of a non-CLOCK::BMAL1 target, *Pmp22*, was measured using quantitative PCR. Data were expressed relative to input DNA. Time-of-day affected non-specific binding (no antibody) to *Cry1* (F_3,23_ = 4.2, p<0.05), *Dbp* (F_3,22_ = 3.9, p<0.05), and *Per1* (F_3,22_ = 3.9, p<0.05, all plotted in both BMAL1 and CLOCK columns), but not that of BMAL1 or CLOCK to the control gene *Pmp22*. Data are presented as mean ± SEM. Grey lines connect double-plotted data, and light grey areas represent 12 h dark periods.
(TIF)

Figure S2 **BMAL1 and CLOCK binding onto the promoter of 4 clock genes in the mouse cerebral cortex.** Mice were sacrificed every 6 hours for 24 hours, and brain cortices were rapidly processed. Chromatin immunoprecipitation (ChIP) was performed using antibodies against BMAL1 and CLOCK or no antibody (negative control), and enrichment of putative promoter sequences of *Cry1*, *Dbp*, *Per1* and *Per2*, and of a non-CLOCK::BMAL1 target, *Pmp22*, was measured using quantitative PCR. Data were expressed relative to input DNA. Time-of-day did not significantly affect non-specific binding (no antibody), neither that of BMAL1 or CLOCK to the control gene *Pmp22*. Data are presented as mean ± SEM. Grey lines connect double-plotted data, and light grey areas represent 12 h dark periods.
(TIF)
