# Supplementary figures and images for: Sleep Loss Reduces the DNA-Binding of BMAL1, CLOCK, and NPAS2 to Specific Clock Genes in the Mouse Cerebral Cortex
Source: PLoS One. 2011 Oct 24;6(10):e26622. doi: 10.1371/journal.pone.0026622 (PMC3200344; doi:10.1371/journal.pone.0026622)

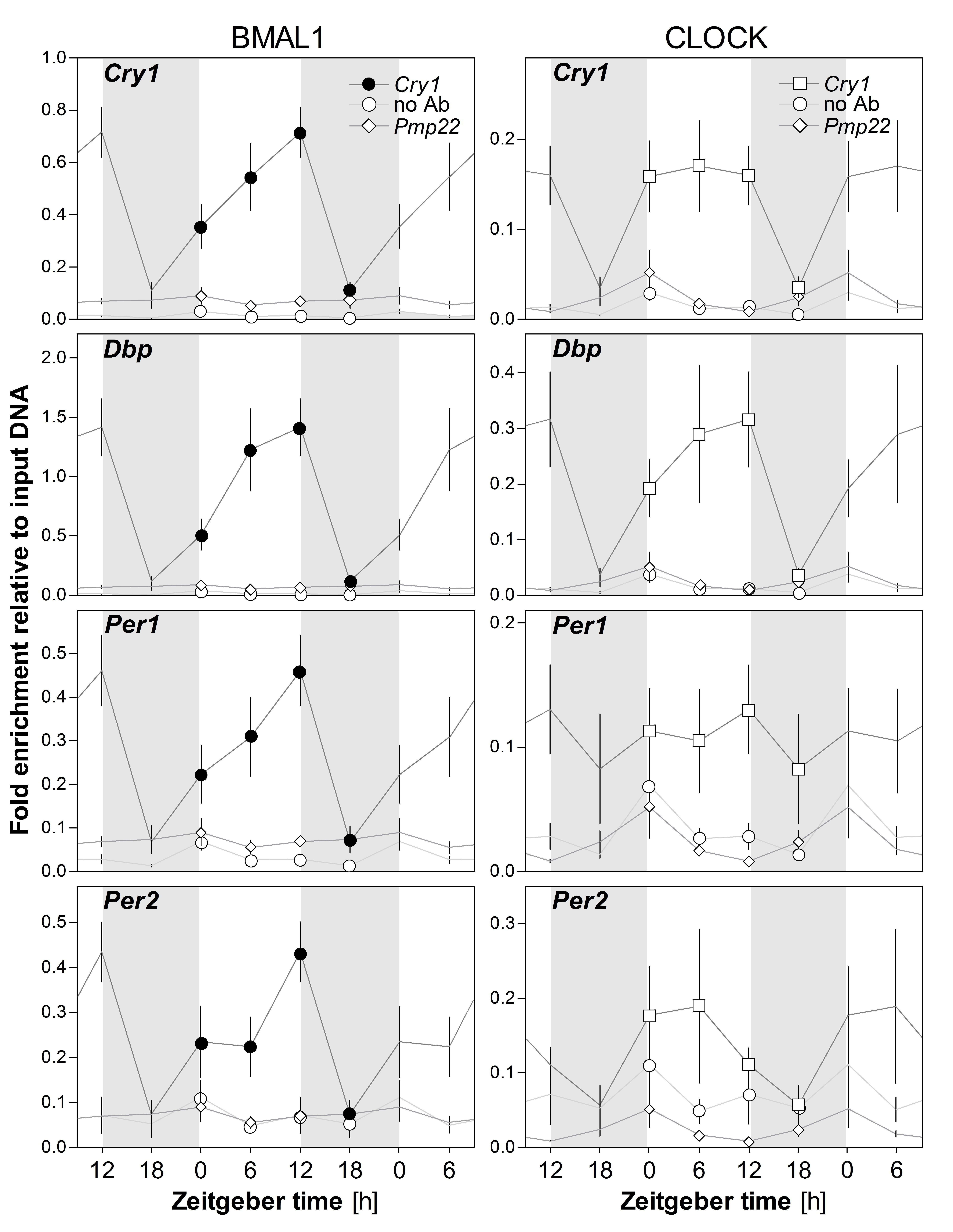

Supplement: Figure S1 — BMAL1 and CLOCK binding onto the promoter of 4 clock genes in the mouse liver. Mice were sacrificed every 6 hours for 24 hours, and livers were rapidly processed. Chromatin immunoprecipitation (ChIP) was performed using antibodies against BMAL1 and CLOCK or no antibody (negative control), and enrichment of putative promoter sequences of Cry1, Dbp, Per1 and Per2, and of a non-CLOCK::BMAL1 target, Pmp22, was measured using quantitative PCR. Data were expressed relative to input DNA. Time-of-day affected non-specific binding (no antibody) to Cry1 (F3,23 = 4.2, p<0.05), Dbp (F3,22 = 3.9, p<0.05), and Per1 (F3,22 = 3.9, p<0.05, all plotted in both BMAL1 and CLOCK columns), but not that of BMAL1 or CLOCK to the control gene Pmp22. Data are presented as mean ± SEM. Grey lines connect double-plotted data, and light grey areas represent 12 h dark periods. (TIF) [file pone.0026622.s001.tif]

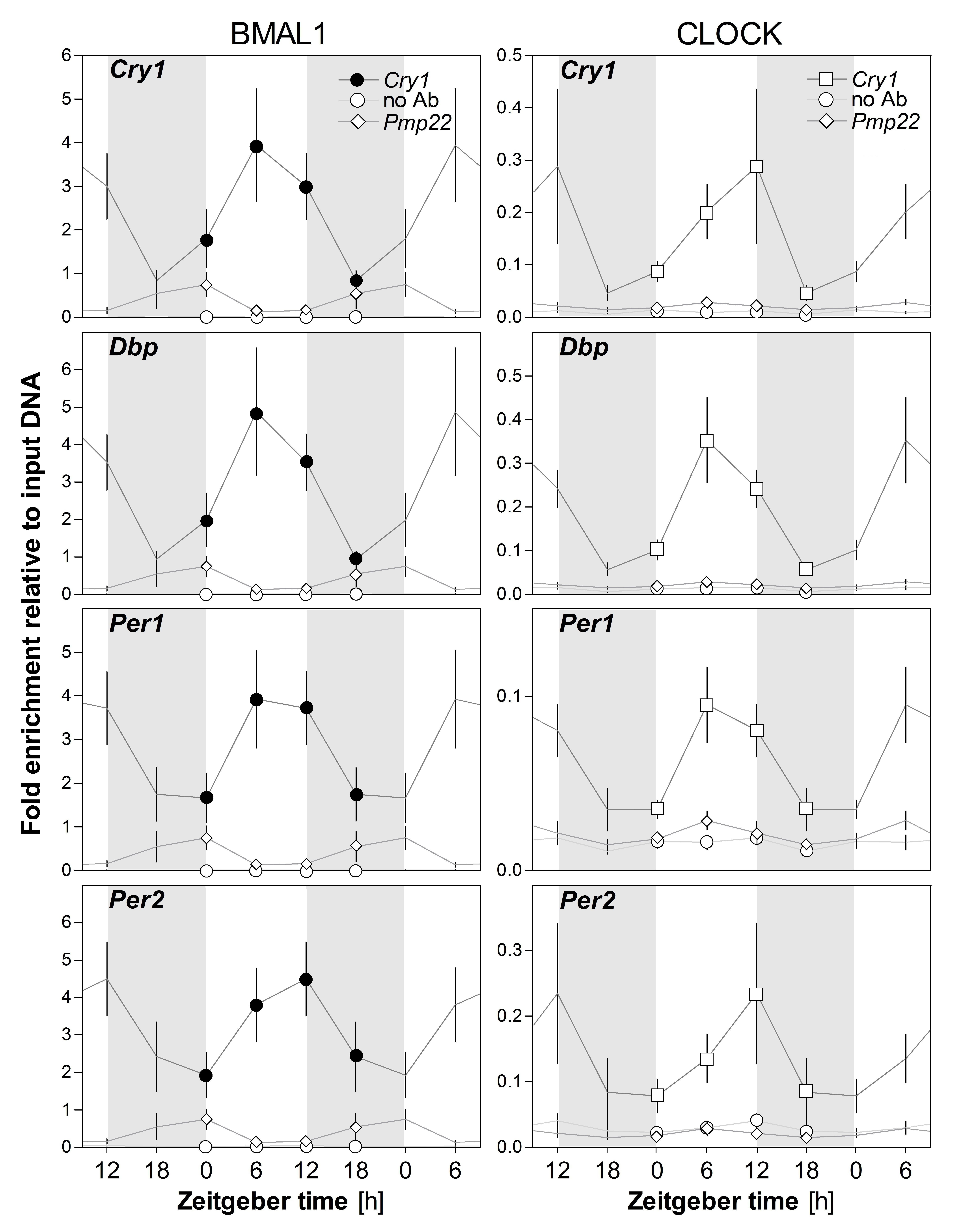

Supplement: Figure S2 — BMAL1 and CLOCK binding onto the promoter of 4 clock genes in the mouse cerebral cortex. Mice were sacrificed every 6 hours for 24 hours, and brain cortices were rapidly processed. Chromatin immunoprecipitation (ChIP) was performed using antibodies against BMAL1 and CLOCK or no antibody (negative control), and enrichment of putative promoter sequences of Cry1, Dbp, Per1 and Per2, and of a non-CLOCK::BMAL1 target, Pmp22, was measured using quantitative PCR. Data were expressed relative to input DNA. Time-of-day did not significantly affect non-specific binding (no antibody), neither that of BMAL1 or CLOCK to the control gene Pmp22. Data are presented as mean ± SEM. Grey lines connect double-plotted data, and light grey areas represent 12 h dark periods. (TIF) [file pone.0026622.s002.tif]
